# Characterizing the Inflammatory Profile of Neutrophil-Rich Triple-Negative Breast Cancer

**DOI:** 10.3390/cancers16040747

**Published:** 2024-02-10

**Authors:** Fatma Al Qutami, Walaa AlHalabi, Aswathy Vijayakumar, Surendra Singh Rawat, Abubakr H. Mossa, Manju Nidagodu Jayakumar, Baila Samreen, Mahmood Y. Hachim

**Affiliations:** 1 Department of Medicine, Mohammed Bin Rashid University of Medicine and Health Sciences, Dubai P.O. Box 505055, United Arab Emirates; fatma.alqutami@mbru.ac.ae (F.A.Q.); walaa.alhalabi@students.mbru.ac.ae (W.A.); aswathy.vijayakumar@students.mbru.ac.ae (A.V.); surendrasingh.rawat@mbru.ac.ae (S.S.R.); baila.samreen@mbru.ac.ae (B.S.); 2 Sharjah Institute for Medical Research, College of Medicine, University of Sharjah, Sharjah P.O. Box 27272, United Arab Emirates; amossa@sharjah.ac.ae (A.H.M.); mjayakumar@sharjah.ac.ae (M.N.J.)

**Keywords:** neutrophiles, triple-negative breast cancer, TNBC, NETosis

## Abstract

**Simple Summary:**

The interaction between the highly aggressive triple-negative breast cancer, and the neutrophils is connected to the poor outcomes of this tumor. Using a wide array of cellular and molecular essays, we found that triple-negative breast cancer cells stressed out the neutrophil-like cells and reduced their viability in cell culture conditions. Furthermore, growing neutrophil-like cells with breast cancer cells enhanced the inflammatory activity of the neutrophil-like cells through the release and expression of chemical or structural inflammatory mediators. Given that cancer cells can exploit the active inflammatory milieu to the advantage of their growth and spread, targeting the crosstalk mechanisms that we illustrated here could help in defining potential therapeutic options in breast cancer management.

**Abstract:**

Breast cancer (BC) is one of the most common types of cancer in women in the United Arab Emirates. Immunogenic tumours, such as triple-negative breast cancer (TNBC), show increased neutrophil infiltration, which is associated with poor prognosis and limited efficacy of immunotherapy. This study aims to investigate in vitro the bidirectional effect of neutrophils on metastatic TNBC (MDA-MB-231) compared to less-metastatic luminal breast cancer (MCF-7) cell lines. We found that BC cells or their conditioned medium (CM) reduced the viability of neutrophil-like cells (HL60). This was supported by increased cellular stress and NETosis in differentiated HL60 cells (dHL60) upon exposure to MDA-MB-231 compared to MCF-7-CM using nucleic acid staining essays. Flow cytometry showed comparable expression of inflammatory markers by polymorphonuclear cells (PMN) when treated with MDA-MB-231-CM and standard polarizing cocktails. Furthermore, MDA-MB-231-CM triggered an inflammatory pattern with evidence of stronger adhesion (CD62L) and degranulation (CD11b and CD66b) phenotypes. The proinflammatory polarization of dHL60 by MDA-MB-231-CM was additionally confirmed by the elevated CD54 expression, myeloperoxidase, and CD11b protein levels, which matched an increased transwell migratory capacity. In conclusion, BC might use neutrophils to their benefit through NETosis and complement system activation, which makes this crosstalk a potential mechanism for understanding tumour progression.

## 1. Introduction

Breast cancer (BC) is one of the most common types of cancer in women, with high incidence rates in postmenopausal women. In the United Arab Emirates, it is the most common cancer and the second leading cause of death due to its aggressive presentation [1,2]. Triple-negative breast cancer (TNBC) is considered highly aggressive due to its mutational burden and immunogenic subtype, containing specific immune infiltrates [3]. Increased neutrophil infiltration in tumour sites is associated with poor prognosis and limited efficacy of immunotherapy [4,5].

Recent studies have shown that tumour-associated neutrophils (TANs) play a role in tumour progression, metastasis, and survival [6]. These TANs are classified into proinflammatory N1 and anti-inflammatory N2 subtypes and/or high- and low-density neutrophils [7]. One mode of TANs action is the deposits of neutrophil extracellular traps (NETs)—a meshwork of chromatin and granule-derived antimicrobial proteins such as histones, myeloperoxidase (MPO), and neutrophil elastase (NE) [8,9,10,11]. NETs have been implicated in metastasis mediation by trapping tumour cells in circulation and depositing them in distant tissues [12]. Studies have shown that increased levels of NET formation (NETosis) are linked to tumour progression and metastasis of several malignancies [13,14].

Furthermore, malignant cells can induce stress on neutrophils by generating reactive oxygen species, activating neutrophils, and releasing other inflammatory mediators which leads to increased NETosis and damage [15]. Since the crosstalk between TNBC and neutrophils is poorly understood, there is a persistent need to understand the exact role of neutrophils in TNBC aggressiveness.

Due to cell-donor variability, several cell lines have been verified as effective models to study the crosstalk between neutrophils and BC. Given the short life span of neutrophils, the neutrophil-like cell line HL60 is an ideal neutrophil model for in vitro studies. The HL60 cell line has been extensively studied and differentiated into a neutrophil-like state in various functional studies such as chemotaxis, NETosis, and phagocytosis [16]. Among the BC cell line models, MDA-MB-231 and MCF-7 cell lines are extensively studied, where MDA-MB-231 represents a highly metastatic poorly differentiated TNBC cell line, while MCF-7 is a low metastatic luminal A BC cell line [17].

In this work, we have used in vitro assays to investigate the role of the bidirectional effect of neutrophils on metastatic TNBC (MDA-MB-231) compared to less-metastatic luminal breast cancer (MCF-7) cell lines. We have further explored the direct effect of BC on neutrophil biology and their antitumour activity presented by NETosis, inflammatory markers expression, degranulation markers, cytokines production, and migration capacity.

## 2. Materials and Methods

### 2.1. Cell Culture and Growth Medium

Breast cancer cell lines used were MCF-7 “Luminal A” and MDA-MB-231 “TNBC”, grown in DMEM/F12cell culture growth medium supplemented with 10% foetal bovine serum (FBS) and 1X Pen/Strep (Gibco, Waltham, MA, USA). HL60 cells were used due to their neutrophil-like properties and cultured in RPMI-1640 GlutaMAX™ media supplemented with 20% FBS and 1X Pen/Strep (Gibco, USA). All cell lines were incubated at 37 °C with 5% CO_2_.

### 2.2. HL60 Cell Line Differentiation

Upon reaching cell growth confluency (0.8–1 × 10^6^ cells/mL), HL60 cells were differentiated into a more neutrophil-like state (dHL60) using 1.3% DMSO (Sigma-Aldrich, St. Louis, MO, USA) and/or 1–5 µM of all-trans retinoic acid (ATRA) (Sigma-Aldrich, USA) added to culture medium for five to seven days at a concentration of 1.5 × 10^5^ cells/mL. The culture medium was renewed every 48–72 h with the addition of differentiating agents, and cell differentiation was confirmed daily using Giemsa stain after day four of treatment. When more than 70% of cells differentiated, cells were transferred into fresh culture media and used for experiments. Differentiated cells were viable for about a week post-differentiation.

### 2.3. Conditioned Media

To obtain conditioned media (CM) of BC (MCF-7 or MDA-MB-231) and HL60 cell lines, cells were cultured up to 70% confluency. The culture medium was replaced with fresh, complete media, and cells were incubated for 24 h before media collection. The whole cell suspension was taken for HL60 (HL60 or dHL60), and cells were separated from the supernatant by centrifugation. The supernatant of both BC and HL60 cell lines was centrifuged for 10 min at 1500 rpm to remove any floating cells and debris. The supernatant was transferred into a new conical tube and stored at −80 °C. Each tube was used only once to avoid freeze-thaw cycles.

### 2.4. Cell Proliferation and Wound Healing Assay Using Conditioned Media

To determine the ideal concentration of BC CM to add to the neutrophil-like cells, a proliferation assay was performed on HL60 cells using MCF-7 CM (used as a control) at increasing concentrations on a 24-well plate at 37 °C in an IncuCyte^®^ (Essen Bioscience, Ann Arbor, MI, USA) for 40 h. Images were recorded every 4 h, and cell confluency was measured using the built-in IncuCyte^®^ software (Machine: Incucyte Zoom 2015A Rev1; Software: Live-Cell Imaging & Analysis Software (https://www.essenbioscience.com/en/resources/incucyte-zoom-resources-support/software-modules-incucyte-zoom/, accessed on 1 March 2023)) and further analysed using GraphPad Prism software 10.

To determine the effect and concentration of both HL60 and dHL60 CM on BC cell lines, a wound-healing assay was performed on the MCF-7 cell line. The MCF-7 cell line was seeded at 4 × 10^5^ cells/well in a 96-well plate, and upon reaching 100% confluency, MCF-7 cells were wounded using IncuCyte^®^ WoundMaker (Sartorius, Göttingen, Germany). Cells were treated with different HL60 and dHL60 CM dilutions and incubated in an IncuCyte^®^ (Essen Bioscience, Ann Arbor, MI, USA) for 54 h, with images of each well recorded every 6 h. Wound width was measured using the built-in scratch wound analysis tool and further analysed using GraphPad Prism software v9.5.1. Controls for the proliferation and wound healing assays included complete media used for BC cell line and HL60 culture.

### 2.5. BC and dHL60 Viability following Coculture

BC cell lines and dHL60 were cocultured in 12-well plates at a ratio of 10:1 BC to dHL60 (4 × 10^5^: 4 × 10^4^ cells) to assess the direct effects of each cell type on one another. All cell lines were supplied with fresh medium, and the medium ratio was 1:1 of BC growth media and dHL60 growth media. Cells were either cocultured immediately at seeding or after BC cells adhered to the cell culture plates. Cell viability was calculated after 24 h using 0.04% trypan blue (Sigma-Aldrich, USA) at a 1:1 ratio, as a percentage of the reference control cells. Controls included cells supplemented with their growth medium and with a 1:1 BC and HL60 growth medium. This was performed to assess if the observed effects were due to the composition of the media or the mutual cellular effect.

### 2.6. NETosis

dHL60 cells were cultured with 100% CM from MDA-MB-231 or 100 ng/mL lipopolysaccharide (LPS) (positive control) and stained with NucBlue™ live ReadyProbes™ reagent (Hoechst 33342) and SYTOX™ Green Ready Flow™ reagent (Invitrogen™, Waltham, MA, USA) for 30 min before they were visualized under a florescent microscope and analysed using Fiji (ImageJ) software (http://imagej.net (accessed on 1 March 2023)). NucBlue is a cell-permeable DNA stain, staining both living and dead cells, while Sytox Green requires a compromised cell membrane to stain and is an indicator of dead or dying cells.

If both stains are positive, it could indicate that neutrophils have ruptured their plasma membrane, and their intracellular contents were released into the extracellular space, which is seen in neutrophils undergoing NETosis. This is another indicator of the N1 subtype. Cells that only stain positive for Sytox green and are negative for NucBlue have either undergone necrosis or late-stage apoptosis, as these cells have a compromised plasma membrane. NucBlue-positive only cells are cells that have undergone some nuclear changes, i.e., chromatin de-condensation, as in the early stages of NETosis [17,18]. Stained cells were quantified using flow cytometry with a calibrated BD FACSAria III flow cytometer (BD Biosciences, San Jose, CA, USA) using a standard configuration with BD FACSDiva software v9.0 (BD Biosciences, San Jose, CA, USA).

### 2.7. Flow Cytometry of Neutrophils Isolated from Whole Blood

Neutrophils were isolated from whole blood from volunteer donors using the MACSxpress^®^ Whole Blood Neutrophil Isolation Kit (Miltenyi Biotec, Bergisch Gladbach, Germany). Five millilitres of whole blood were used to isolate neutrophils according to manufacturer protocol, and the purity was checked using a Giemsa stain. An amount of 1 × 10^6^ cells were seeded per well with 500 µL of media and were stimulated with one ofMCF-7, MDA-MB-231 CM, or a neutrophil polarization cocktail and incubated for 30 min at 37 °C.

Cells were either polarized to a proinflammatory (N1) or anti-inflammatory (N2) state using LPS, IFN-gamma, IFN-beta, TGF-beta, IL10, PGE_2_, G-CSF, L-lactate, and Adenosine as described by Ohms et al. [19] (Table 1). Cells were also stained with CD11b, CD15, CD66b, CD62L, CD54, and CD182 and fixed before characterization using BD FACSAria III flow cytometer (BD Biosciences, San Jose, CA, USA).

### 2.8. Myeloperoxidase (MPO) and Neutrophile Elastase (NE) Expression by dHL60

dHL60 cells were polarized with an N1 and N2 polarization cocktail or BC CM for 30 min and 4 h. The cells were also stimulated using the components of each polarization cocktail individually for 30 min and 4 h. The supernatant was separated from cells, and ELISA was performed on samples using the Human MPO SimpleStep ELISA^®^ kit (ab272101; Abcam, Cambridge, UK) and the Human Neutrophil Elastase ELISA^®^ Kit (ab270204; Abcam, UK). The kit protocol was used, samples were diluted 1:2 per instructions, and OD was measured at 450nm using a HIDEX Sense plate reader (Turku, Finland). The concentration of MPO and NE was calculated using a standard curve, and the cell culture medium was used as blank.

### 2.9. Flow Cytometry of dHL60

The dHL60 cells were subjected to treatment with 50% CM obtained from either MCF-7 or MDA-MB-231 cell lines for 24 h, with or without the addition of 100 ng/mL of LPS (InvivoGen, San Diego, CA, USA). LPS is a known inducer of inflammation and was utilized as a control to polarize neutrophils into the inflammatory subtype. Following treatment, the cells were harvested and stained using an antibody of CD54 following the manufacturer’s instructions (BD Biosciences, San Jose, CA, USA). The samples were incubated for 30 min, washed, and subjected to marker expression analysis by acquiring samples using either a calibrated BD FACSAria III flow cytometer (BD Biosciences, San Jose, CA, USA) using a standard configuration with BD FACSDiva software v9.0 (BD Biosciences, San Jose, CA, USA) or an Amnis CellStream (Luminex Corp, Austin, TX, USA) flow cytometer with CellStream software (CellStream Analysis 1.3.384). A total of 10,000 events were collected for each sample, and the data analysis was performed using FlowJo software (FlowJo™ v10, FlowJo LLC., Ashland, OR, USA).

### 2.10. Trans-Well Migration Assay

Neutrophil migration was tested using a 3.0 µM Falcon transparent PET membrane (Cat no. 353096; Corning, Glendale, AZ, USA). Three hundred thousand cells were incubated in the upper chamber of each membrane with a normal culture medium. In the lower chamber, control samples included the HL60/dHL60 culture medium and IL-8 chemoattractant. The CM of both MCF7 and MDA-MB-231 were added in lower chambers to assess their chemo-attractive capabilities on dHL60. Cells were incubated at 37 °C for one hour before migration was measured. Media remaining in upper chambers were transferred into new wells, and inserts were washed with PBS, fixed in 4% PFA, stained with Giemsa, and counted. The media of the lower chamber and upper chamber were stained with NucBlue™ live ReadyProbes™ reagent (Hoechst 33342), a flat plate reader measured fluorescence, and images were taken of cells under a fluorescent microscope.

### 2.11. Western Blotting

BC and dHL60 cell lines were cocultured in 15cm plates at a ratio of 10:1 BC to neutrophils (1.5 × 10^7^: 1.6 × 10^6^ cells) and incubated at 37 °C for 24 h. dHL60 cells were centrifuged at 200× *g* for 7 min and lysed with a 1× cell lysis buffer (Cat no. 9803S; Cell Signaling Technology, Danvers, MA, USA). BC cells were lysed directly on the plate, and the lysate was collected via scraping. Samples were prepared with a 1× Laemmli buffer (Cat no. 1610747; Bio-Rad Laboratories, Hercules, CA, USA) and incubated at 95 °C for 5 min. A total of 18 µg of the sample was loaded on a 10% SDS-Polyacrylamide gel, and a 120 V power supply was applied for one hour. Protein was transferred to a nitrocellulose membrane using BioRad Trans-blot apparatus (Bio-Rad Laboratories, USA). While shaking, the membrane was blocked with 5% bovine serum albumin (BSA) in 1× phosphate buffer saline with Tween 20 (PBST) for one hour at room temperature. The membrane was incubated with primary antibodies overnight at 4 °C with shaking. After primary antibody binding, the membrane was washed for 5 min with 1× PBST three times and then incubated with Anti-Rabbit HRP secondary antibodies at 1:2000 dilution (Cat no. 7074S; Cell Signaling Technology, USA) for one hour at room temperature while shaking. Then, the blot was washed thrice with 1× PBST for 5 min and developed with an Enhanced Chemiluminescence (ECL) substrate (Cat no. 1705061; Bio-Rad Laboratories, USA). Primary antibodies used included CD11b at 1:1000 dilution (ab224805; Abcam, UK), and GAPDH (MA116757; Invitrogen, USA) as a housekeeping primer with 1:2000 dilution. The Novex sharp pre-stained protein standard (LC5800; Invitrogen, USA) was used as a ladder with a weight of 3.8 to 260 kDa.

### 2.12. Statistical Analyses

All statistical analyses were performed on GraphPad Prism software v9.5.1 or R studio (version 4.2.2). One-way and two-way ANOVA and unpaired *t*-tests were carried out as appropriate. Results were considered statistically significant with *p* < 0.05. For flow cytometry data, results were analysed using CytoExplorerR in R Studio (R version 4.2.2) and FlowJo software (FlowJo™ v10, FlowJo LLC., Ashland, OR, USA). Gating data was then imported into GraphPad Prism software v9.5.1 for further statistical analysis.

## 3. Results

### 3.1. HL60 Cells Differentiated into a Neutrophil-like State

**Figure 1:**
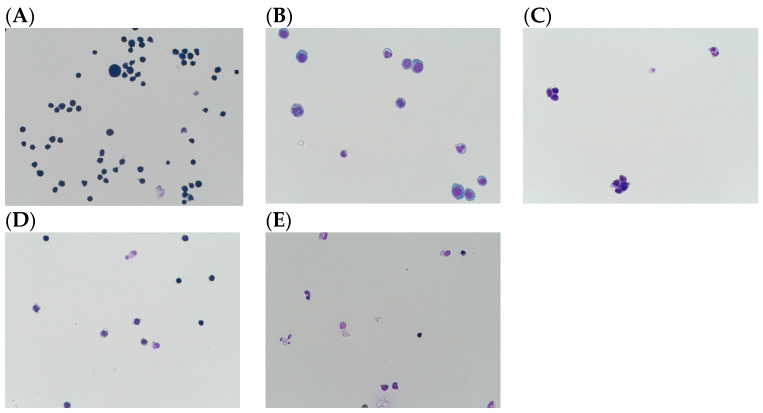
HL60 cells differentiation by 6 days in different conditions: (**A**) HL60 undifferentiated (Control), (**B**) 1 µM ATRA-treated cells show large, meta-myelocyte morphology, (**C**) 5 µM ATRA-treated cells show small clumping banded cells, (**D**) 1.3% DMSO-treated cells show slower differentiation, (**E**) combination of 1 µM ATRA and 1.3% DMSO caused banded neutrophils to hyper-segmented morphological differentiation, 40× magnification.

HL60 cells were stained with a Giemsa stain daily after 72 h of differentiation for a period of 6 days. Before differentiation, cells were small, circular, and closely resembled myelocytes, heavily staining blue. After 72 h, the cells became larger in size. At 96 h, cell morphology started to change, with some bands beginning to appear. By day 6 (144 h post-differentiation), most of the cells were differentiated (Figure 1), with varying levels between the conditions. Cells treated with 1 µM ATRA had large, meta-myelocyte morphology (Figure 1B), while 5 µM ATRA-treated cells had smaller-looking clumping cells but were more banded (Figure 1C). DMSO-treated cells required a longer time to undergo differentiation, as there were fewer differentiated cells than ATRA, though differentiated cells had a banded neutrophil morphology (Figure 1D). The combination of ATRA and DMSO induced the most change, with cells exhibiting differentiating properties. The cells treated with both agents range from banded neutrophils to hyper-segmented neutrophils (Figure 1E).

### 3.2. The CM Caused a Reciprocal Inhibition of Proliferation in HL60 and MCF-7 Cells

**Figure 2:**
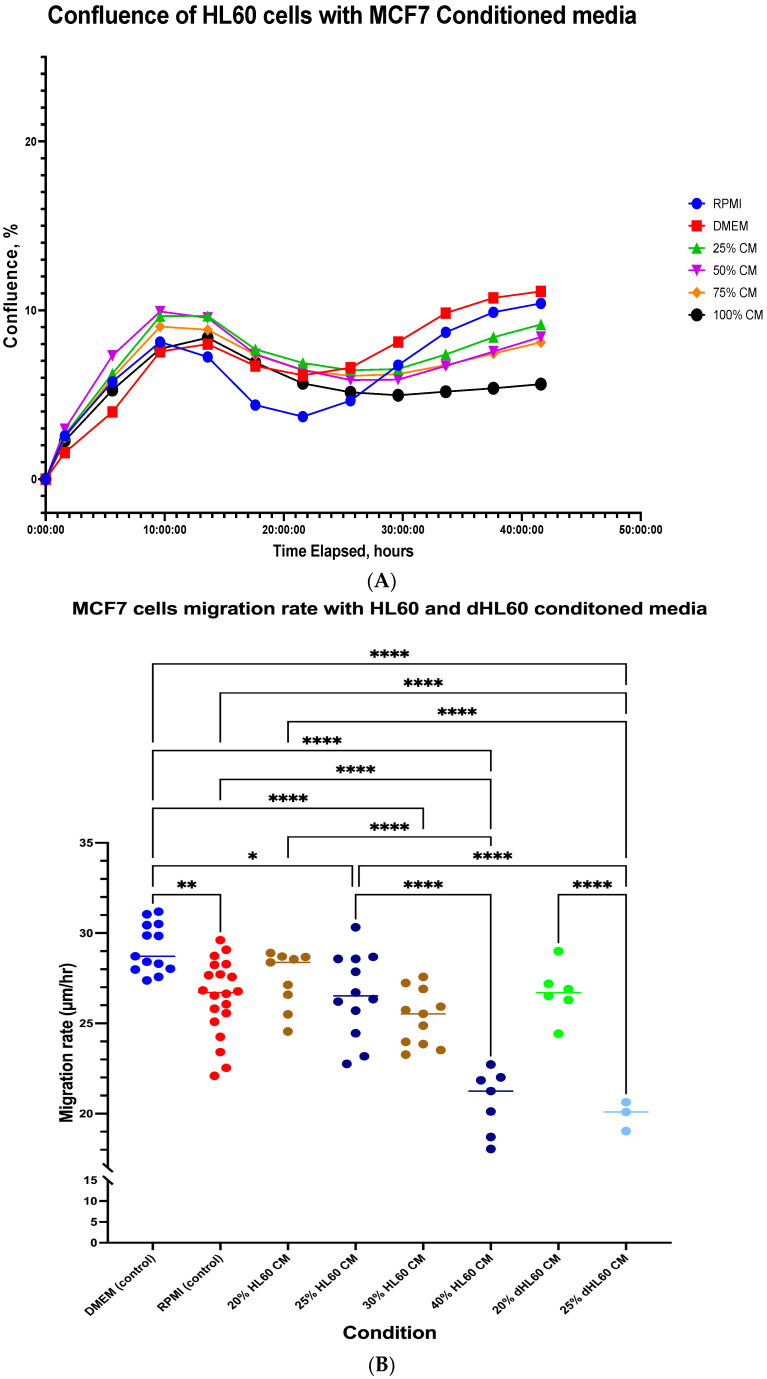
The effect of culturing dHL60 or MCF-7 cells in CM on proliferation. (**A**) The rate of confluence of HL60 was affected by both time and concentration of MCF-7 conditioned media. (**B**) The migration rate of MCF-7 is different when comparing HL60-CM and dHL60-CM treated groups, with a low concentration of dHL60 (25% CM) eliciting similar effects to a medium concentration (40% CM) of HL60. The statistical significance was calculated using one-way ANOVA and is denoted by the following: **** = *p* < 0.0001; ** = *p* < 0.01; * = *p* < 0.05.

To confirm the effect of BC cells on the viability and proliferation of neutrophils, HL60 cells were incubated with different concentrations of MCF-7 CM. MCF-7 cells were selected to serve as a comparative control to MDA-MB-231 cells. This selection was based on MCF-7 low metastatic capabilities, and therefore, we needed to identify the minimum concentration of CM to illicit an effect on the HL60 cell lines. Pre-differentiation cells were used because dHL60 cells exit the proliferative cell cycle with a minor subset of incompletely differentiated cells that retain their proliferative capacity. Results showed that the proliferation of HL60 was both dose- and time-dependent (Figure 2A). The cells started proliferating, as seen in an increase in confluence until 10 h, then proceeded to decrease before increasing once again, reflecting a time-dependent effect. At the endpoint of 40 h, the cells exhibited dose-dependent differences in proliferation, with 100% CM having the least increase in confluence compared to controls, while 25, 50, and 75% CM were close in effect and therefore not statistically different as inferred by one-way ANOVA analysis (Figure 2A).

Two-way ANOVA multiple comparisons using a full model showed no differences between RPMI media and all other conditions, including DMEM. However, when comparing DMEM with MCF7 CM, some differences were detected. At the 5-hr time point, there was a difference between DMEM, 50% CM (3.34%, *p* = 0.0123), and 75% CM (2.017%, *p* = 0.0265). This is the only time point a statistical difference was observed between the conditions. This can semi-reflect on neutrophil activity, as neutrophils exhibit the highest activity at the earliest time point of exposure to pathogens. However, as these cells were not differentiated, their anti-inflammatory activity might not resemble neutrophils, but instead resemble proliferative cells in the bone marrow before neutrophil maturation and release into the bloodstream.

On the other hand, comparing the CM of both HL60 and dHL60 on the BC cell line showed that dHL60 CM had an inhibitory effect on MCF-7 in a dose-dependent manner. The migration rate of MCF-7 was affected by both HL60 and dHL60 CM (Figure 2B). Between the two controls, RPMI 1640 media had a slightly, yet statistically significant, lower migration rate, with a mean difference of 2.750 µM/h (*p* = 0.0011), indicating a small effect of media on the migration of MCF-7.

Comparing DMEM control (29.17 µm/h) to HL60 CM treatments, there was a significant decrease in MCF-7 migration rate with a mean difference of 2.561, 3.865, and 8.595 µm/h for 25% HL60 CM (26.61 µM/h, *p* = 0.0181), 30% HL60 CM (25.31 µm/h, *p* < 0.0001), and 40% HL60 CM (20.67 µm/h, *p* < 0.0001), respectively. At the same time, lower concentrations of dHL60 CM had more impact on the MCF-7 migration capacity, with a mean difference of 2.454 and 9.256 µm/h in 20% dHL60 CM (26.72 µm/h, *p* = 0.1837) and 25% dHL60 CM (19.92 µm/h, *p* < 0.0001), respectively (Figure 2B).

Moreover, when assessing the effects of CM in reference to RPMI control (26.42 µm/h), similar patterns were shown, with 40% HL60 CM having a mean difference of 5.754 µm/h (*p* < 0.0001) and 25% dHL60 CM having a mean difference of 6.504 µm/h (*p* < 0.0001). Even though almost half the concentration of dHL60 (25%) was able to show the same difference as HL60 CM (40%), there was no significant difference between the two conditions (mean difference = 0.7514 µm/h, *p* > 0.9999). Furthermore, our results showed that at 20 and 25% of CM, dHL60 exhibited more inhibitory properties compared to HL60 alone. These results indicate that neutrophils exhibit anti-proliferation activity in luminal cell lines, with a stronger effect of cytokines produced by the differentiated neutrophile-like cells.

### 3.3. Coculture Reduced dHL60 Viability Depending on Tumour Type and Adherence

**Figure 3:**
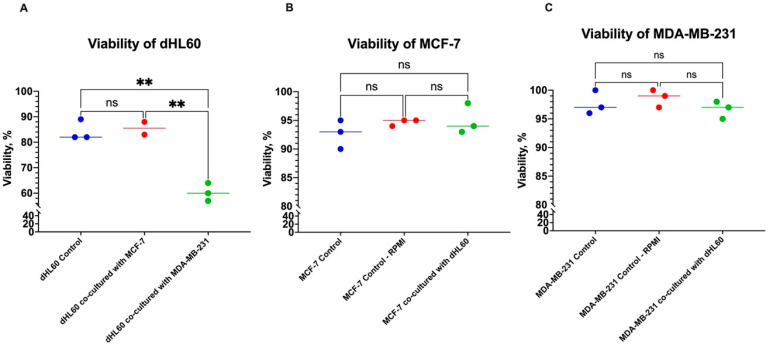
The viability of cells cocultured at seeding for 24 h. (**A**) There is a viability decrease in dHL60 cocultured with MDA-MB-231 of more than 20% compared to dHL60 controls. However, the dHL60 cell line does not affect the viability of (**B**) MCF-7 and (**C**) MDA-MB-231 BC cells. The statistical significance was calculated using one-way ANOVA and is denoted by ** = *p* < 0.01; ns = insignificant.

To further assess the direct effect of neutrophil-BC interactions on cell viability, cell lines were co-cultured in a 12-well plate at a ratio of 10:1 BC cell line to dHL60 for 24 h (Figure 3). Some studies have shown that circulating tumour cells use neutrophils to increase their viability and aid in metastasis. Therefore, dHL60 was co-cultured before and after BC cells were able to establish extracellular matrix (ECM), as well as before that establishment, to mimic the BC environment [29].

BC cells co-cultured while seeding (before the establishment of ECM) were not affected by the neutrophil cell line (Figure 3B & 3C). However, dHL60 viability decreased in cells cocultured with the TNBC cell line MDA-MB-231 (Figure 3A). There was a 24% decrease in viability between dHL60 controls and those co-cultured with MDA-MB-231 (*p* = 0.0013), while there was a 1.167% increase in those cultured with MCF-7 (*p* = 0.9383) (Figure 3A). Comparing the viability of dHL60 co-cultured with both BC cell lines showed a 25.17% difference (*p* = 0.0017), with cells co-cultured with MDA-MB-231 having lower viability in comparison to MCF-7. This decrease in viability can be explained by MDA-MB-231 cells being able to induce cell death in dHL60.

**Figure 4:**
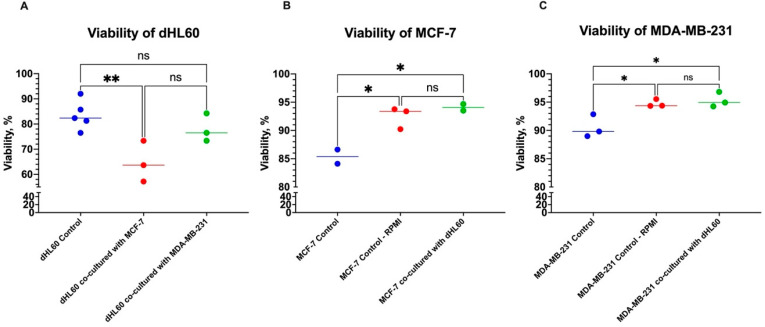
The viability of cells cocultured after BC cell lines were left to adhere to the plate for 24 h. (**A**) The viability of dHL60 decreased in MCF-7, as opposed to MDA-MB-231. There was no effect on the viability of cocultured (**B**) MCF7 and (**C**) MDA-MB-231 BC cells; rather, there was a significant increase in viability. The statistical significance was calculated using one-way ANOVA and is denoted by ** = *p* < 0.01; * = *p* < 0.05; ns = insignificant.

Interestingly, when the BC cells were left to adhere to culture vessels and establish an ECM, the effect on dHL60 was contradictory (Figure 4A). In dHL60 cells cocultured with MCF-7, there was an 18.85% decrease in viability (*p* = 0.0094) compared to controls, while MDA-MB-231 cells did not cause a statistically significant decrease in dHL60 viability (5.553%, *p* = 0.4928).

Both MCF-7 and MDA-MB-231 also showed improved viability in the RPMI growth medium and the dHL60 CM (Figure 4B,C). This growth could be attributed to the composition of the growth medium used. The dHL60 growth medium (RPMI-1640 GlutaMAX™) was supplemented with 20% FBS, as opposed to the BC growth medium (DMEM/F12) with 10% FBS. This increase was rather significant, as MCF-7 cell viability increased by 7.088% (*p* = 0.0215) (Figure 4B), and in MDA-MB-231, the viability increased by 4.193% (*p* = 0.0290) (Figure 4C).

**Figure 5:**
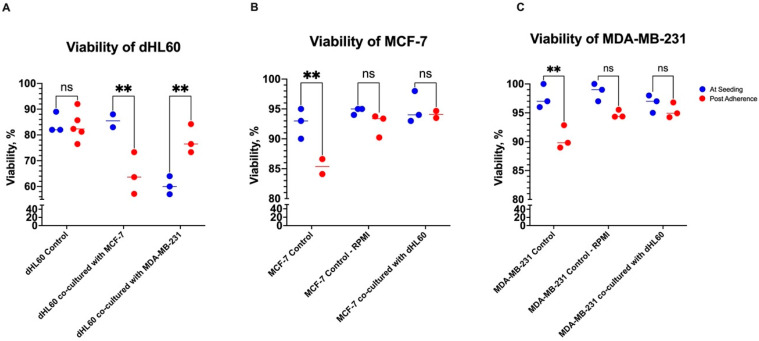
Comparing the viability of cells between co-culturing at seeding and post adherence of BC. (**A**) Cocultured dHL60 viability changes were dependent on the tumour type and adherence. (**B**) MCF-7 and (**C**) MDA-MB-231 BC cells did not show a change in viability when cocultured with dHL60 at-seeding, yet their viability was improved by coculture post-adherence. The statistical significance was calculated using one-way ANOVA and is denoted by ** = *p* < 0.01; ns = insignificant.

These results illustrate BC’s direct effects on neutrophils, which appear to depend on the tumour type and classification. Furthermore, when the cells were co-cultured at seeding, they behaved differently before any form of adherence than cells where BC was allowed to adhere to the culture vessel first (Figure 5). This can be seen by the increase in BC viability under the influence of the RPMI media—both control and CM—in the post-adherence group compared to the at-seeding group (Figure 5B,C). These differences can be attributed to the gene and protein expression between different cancer cell states, releasing distinct molecules or signals in each condition. For example, BC cells can release or block some adhesion molecules during certain stages of cancer to aid tumour progression [30].

### 3.4. MDA-MB-231 CM Induced NETosis in dHL60 Similar to LPS

To understand if the viability of dHL60 was affected by BC cells and CM, the level and mode of cell death were assessed by culturing dHL60 in MDA-MB-231 CM, MCF-7 CM, and LPS. Through unique patterns and combinations of NucBlue and Sytox Green stains, the mode of cell death can be identified as NETosis, apoptosis, or necrosis. dHL60 cells that are positively stained with NucBlue and Sytox Green are cells that are undergoing NETosis.

**Figure 6:**
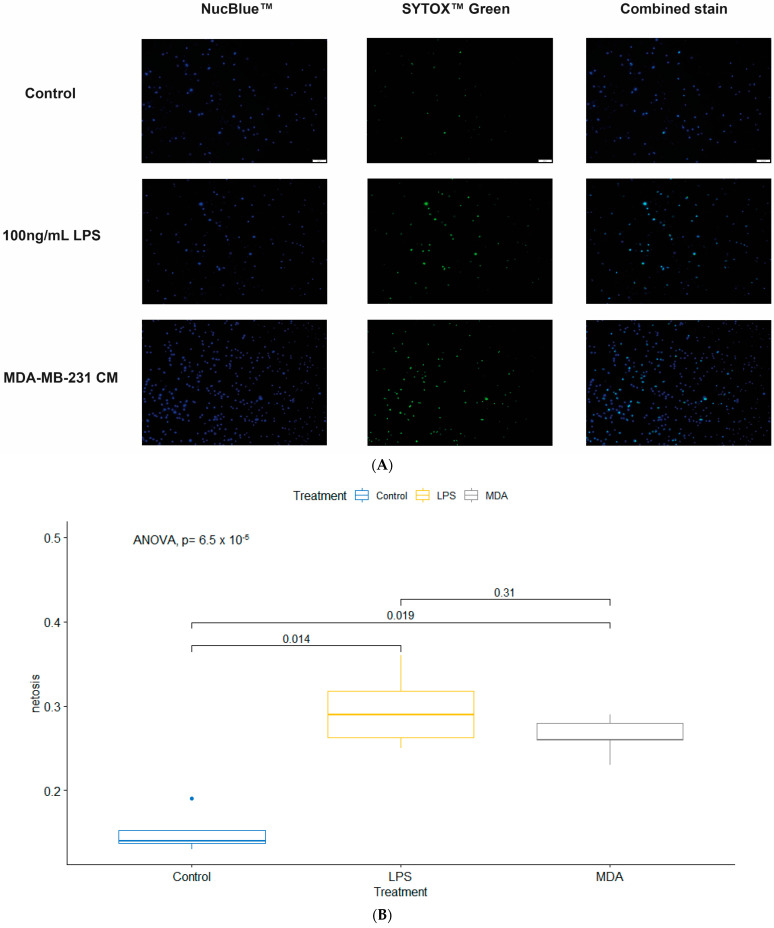
dHL60 underwent NETosis upon treatment with CM. (**A**) Fluorescent imaging of dHL60 shows that cells stained for both NucBlue (DNA stain) and SYTOX Green (live/dead DNA stain) are cells undergoing NETosis. Scale bar is 50 μm (**B**) Percentage of dHL60 cells undergoing NETosis within 30 min of being treated with either LPS or 100% MDA-MB-231 CM.

Lipopolysaccharide (LPS) is a known inducer of inflammation and therefore is an ideal control. The percentage of cells that were stained double positive in the presence of LPS was higher than that of controls, which is in line with the inflammatory effect of LPS (Figure 6). Interestingly, MDA-MB-231 CM also showed a significantly higher level of NETosis than the control, and there was no difference between MDA-MB-231 CM and LPS. This indicates that secreted particles in MDA-MB-231 CM can induce NETosis comparable to LPS.

**Figure 7:**
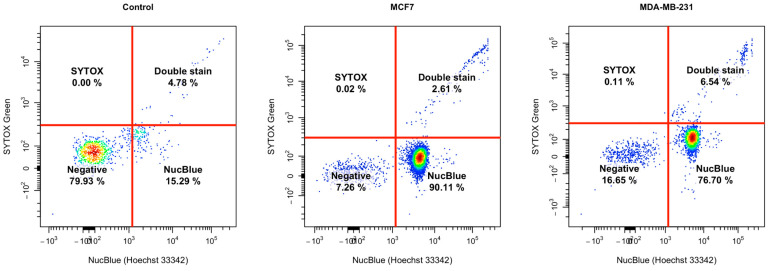
Flow cytometry shows distinct populations in dHL60 treated with MCF-7 and MDA-MB-231 CM. Expression of Sytox Green and NucBlue show that the majority of cells are NucBlue positive following CM treatment, and MDA-MB-231 has more Sytox Green positive cells compared to MCF-7.

To further quantify the number of cells in each group, we used flow cytometry to quantify the expression of cells with their respective stains (Figure 7). Interestingly, there are three distinct populations of cells based on their Sytox and NucBlue expression, with some notable shifts between the cells (Figure 7). Data showed that most of the cells stained NucBlue positive and very few stained Sytox positive, indicating there were fewer dead cells, though the increase in the NucBlue between BC CM and controls could indicate that there was a population shift due to nuclear changes to the cell.

**Figure 8:**
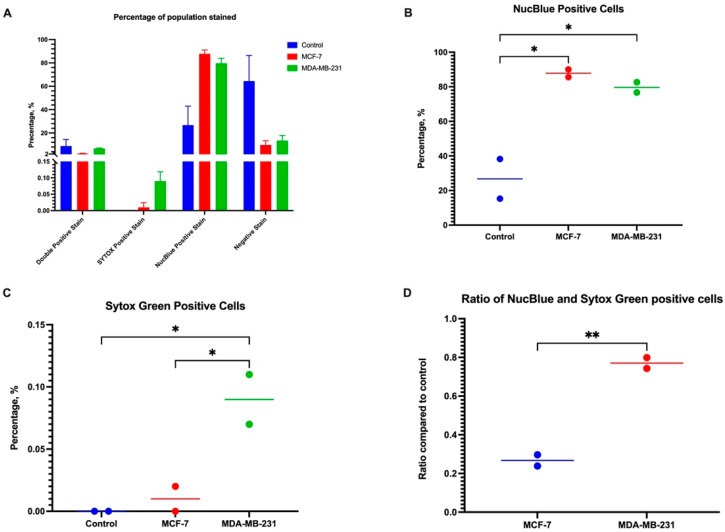
Statistical analysis shows that there was a difference in the stain uptake of duplicate readings. (**A**) A comparison between each stain in each treatment condition. (**B**) NucBlue-positive cells were higher in BC-CM-treated dHL60. (**C**) The MDA-MB-231 CM group had the highest percentage of Sytox Green positive cells; therefore, there were more necrotic and apoptotic cells. (**D**) MDA-MB-231 CM-treated cells have a higher double–positive ratio compared to MCF-7. The statistical significance was calculated using one-way ANOVA and is denoted by ** = *p* < 0.01; * = *p* < 0.05.

Statistical analysis (Figure 8) of the NucBlue positive population showed that the number of positively stained dHL60 cells in MCF-7 CM and MDA-MB-231 CM were 61.01% (*p* = 0.0172) and 50.92% (*p* = 0.0256) higher compared to controls. No difference existed between cells stimulated with MCF-7 and MDA-MB-231 CM (*p* = 0.7132).

In the Sytox-positive population, there was a 0.09% difference between dHL60 control and MDA-MB-231 CM (*p* = 0.0323), and a 0.08% difference between MCF-7 and MDA-MB-231 (*p* = 0.0442). This suggests that even though both types of CM exhibited similar changes to the DNA of dHL60, MDA-MB-231 CM induced more detrimental stress on the cells, leading to their death. The ratio of the double positive population to the control was calculated, and there was a significant difference ($\bar{d}$ = 0.5034 ± 0.04018, *p* = 0.0063) between MCF-7 CM ($\bar{x}$ = 0.2976) and MDA-MB-231 CM ($\bar{x}$ = 0.7710), indicating that MDA-MB-231 induced more stress on dHL60.

### 3.5. TNBC CM and N1 Polarizing Medium Caused Similar Expression of Inflammatory Markers on Polymorphonuclear Cells (PMNs)

Flow cytometric analysis was performed using PMNs isolated from whole blood and stimulated with either MCF-7 CM, MDA-MB-231 CM, N1, or N2 polarization cocktails to assess the expression of inflammatory markers that induce different types of death in neutrophils.

**Figure 9:**
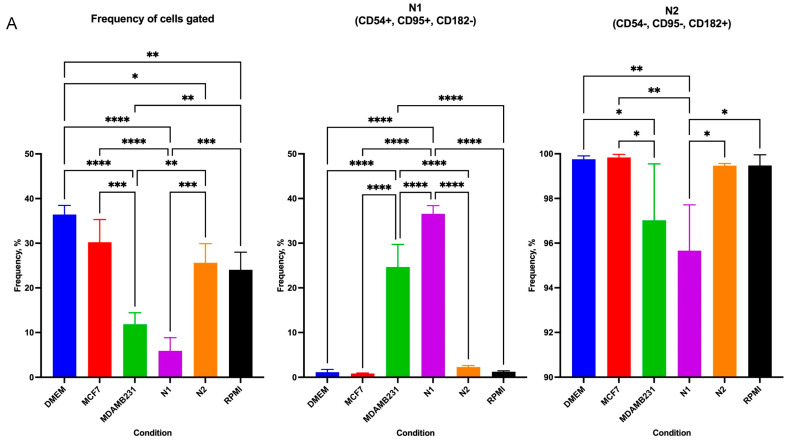

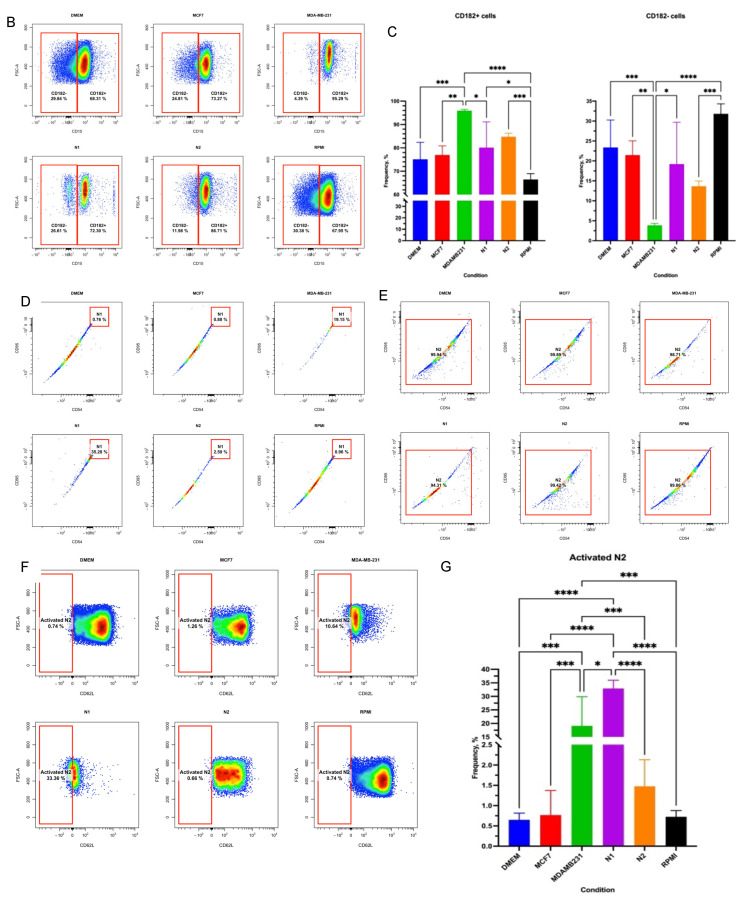

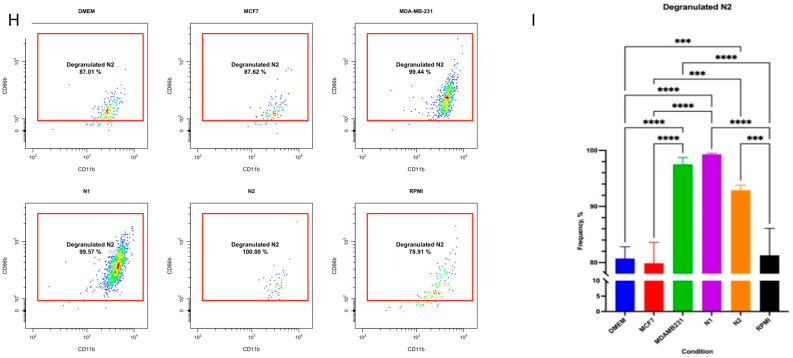
Flow cytometric analysis of neutrophils isolated from whole blood and treated with BC CM or polarizing cocktails. (**A**) frequency of gated populations, N1 and N2 cells show different numbers of cells between MDA-MB-231 and N1 polarized cells compared to other cells. (**B**) The dot plot shows shifting populations based on CD182 expression. (**C**) Statistical analysis shows a significant difference in CD182+ and CD182- populations in MDA-MB-231 CM and N1 polarized cells. (**D**) CD182- population was mostly in N0 state, with MDA-MB-231 having a higher N1 population. (**E**) CD182+ population was mostly in the N2 state with no differences between conditions. (**F**) N2 cells are activated differently between conditions, with (**G**) statistical analysis showing MDA-MB-231 and N1-polarized cells having the highest level of N2 activation. (**H**) From the activated N2 population, most are degranulating, with (**I**) the MDA-MB-231 and N1-polarized cell exhibiting similar degranulation as N2-polarized cells. The statistical significance was calculated using one-way ANOVA and is denoted by ****= *p* < 0.0001; *** = *p* < 0.001; ** = *p* < 0.01; * = *p* < 0.05.

MDA-MB-231-CM and N1-polarized cells exhibited similar patterns and features. These features include clumping or having an irregular shape when they undergo NETosis; therefore, these cells do not appear in the gated single-cell neutrophil populations. This lack of single cells was significant compared to other conditions, with MDA-MB-231 CM having a frequency of 11.84% gated cells, and N1-polarized cells a frequency of 5.898% gated cells, whereas DMEM control, RPMI control, N2-polarized and MCF-7 CM stimulated cells had a frequency of 36.435%, 24.05%, 25.60%, and 30.22%, respectively (Figure 9A).

N1-polarized cells showed a low CD15+ count, a marker used to identify neutrophil populations within PMNs (Figure 9B). In all conditions, most of the cells were CD15+ within the gated cell except for N1 polarized cells. The frequency of CD15+ cells in N1-polarized cells (52.83%) was significantly lower compared to the DMEM control (95.83%, *p* < 0.0001), RPMI control (95.50%, *p* < 0.0001), N2-polarized cells (94.35%, *p* < 0.0001), MDA-MB-231 CM (82.27%, *p* = 0.0003), and MCF-7 CM (94.97%, *p* < 0.0001). Possibly, this was observed because in N1-polarized cells, only 5.898% of the cells were within the single cell gate, suggesting that mature neutrophils have undergone a form of cell death due to the inflammatory stresses of the polarizing agents. Furthermore, studies have suggested that neutrophils isolated from whole blood with a low CD15 expression on their surface are associated with immature neutrophils [31,32].

N1 and N2 subpopulations of neutrophils can be identified using a combination of markers. N1 cells are classified as CD54^High^/CD95^High^/CD182^Low^, while N2 cells are CD54^Low^/CD95^Low^/CD182^High^. Cells were initially identified based on CD182 expression, followed by CD54 and CD95 [Figure 9]. Flow cytometric visualization of CD15+ with FSC-A showed shifting populations, with N1-polarized cells having two distinct populations (CD182^+^ = 80.1%, CD182^−^ = 19.2%) and MDA-MB-231 with a more singular population (CD182^+^ = 95.93%, CD182^−^ = 3.85%) [Figure 9B]. High levels of CD182 are used as a marker for chemotaxis, neutrophil activation, and angiogenesis in sites of inflammation [33,34]. However, CD182 is combined with CD54 and CD95 to identify whether cells are polarized or not completely differentiated yet. This appears to be the case with many cells, as they were CD182+ but had low CD54 and CD95, therefore, they were in a N0 state.

In CD182^low^ cells, there was a low number of CD54+/CD95+ cells, showing a low N1 population in all subtypes (Figure 9A,D). However, there was more than a ten-fold increase in the N1 populations in the N1-polarized and the MDA-MB-231 CM compared to other conditions, in all of which the *p* value was <0.0001. Furthermore, there was a slight difference between MDA-MB-231 CM and N1-polarized cells, with MDA-MB-231 CM having 11.94% less than N1-polarized cells (*p* < 0.0001).

On the other hand, the CD182^high^ population mostly expressed CD54^low^ and CD95^low^, indicating that, in most conditions, over 90% of the CD182+ cells were N2 (Figure 9A). Although there was a high frequency of N2 population in the N1-polarized condition, the sample had significantly lower N2 populations in the overall scheme. Interestingly, MDA-MB-231 CM followed a similar pattern to the N1-polarized sample and was significantly less than the controls, N2-polarized cells, and MCF-7 CM. This indicates that MDA-MB-231 CM could elicit responses similar to that of N1-polarized cells (Figure 9A).

Following the identification of neutrophil subtypes, it is important to understand the activation status of neutrophils, which define their role in the surrounding inflammation. CD62L (L-selectin) is an adhesion marker that can identify active neutrophils. Upon neutrophil contact with an inflammatory pathogen, the cells shed CD62L, initiating the inflammatory process and activating neutrophils [35]. In the N1 subpopulation, 58.01%, 62.47%, 68.13%, 53.68%, 42.67%, and 65.39% of cells were activated in DMEM control, RPMI control, N1-polarized, N2-polarized, MCF-7 CM, and MDA-MB-231 CM, respectively; all of which were not statistically different. But in the N2 subpopulation, there were significant changes in neutrophil activation, with 0.65%, 0.72%, 32.94%, 1.48%, 0.77%, and 19.1% in DMEM control, RPMI control, N1-polarized, N2-polarized, MCF-7 CM, and MDA-MB-231 CM, respectively (Figure 9E,F). The increase of activated N2 between control samples and MDA-MB-231 CM was 20-fold, with MDA-MB-231 CM having 17.62% more activated N2 cells than the N2-polarized population (*p* = 0.0005).

In activated neutrophils, the granules and neutrophil degranulation level play an important role in neutrophil function. CD11b (Mac-1) and CD66b (CECAM8) should be positively expressed in degranulating neutrophils. CD11b expression and CD62L cleavage indicate primed neutrophils, and the surface density of CD11b increases during the process. CD66b expression is linked with the activation of secondary granules, and increased expression is associated with increased inflammation [35,36]. Our results showed that the activated N1 population does not undergo high levels of degranulation, where 15.89%, 16.63%, 15.2%, 11.22%, 17.84%, and 13.71% of DMEM control, RPMI control, N1-polarized, N2-polarized, MCF-7 CM, and MDA-MB-231 CM were degranulating, respectively. However, N2 cells had an increased degranulation rate, with 80% or more of the cells showing CD11b+/CD66b+. Interestingly, both N2 from the N1-polarized and MDA-MB-231 CM treated cells exhibited high levels of degranulation, similar to the N2-polarized cells. MCF-7 CM did not cause cells to degranulate and were of similar levels to controls; this points towards MDA-MB-231 CM having a more inflammatory effect on cells.

**Figure 10:**
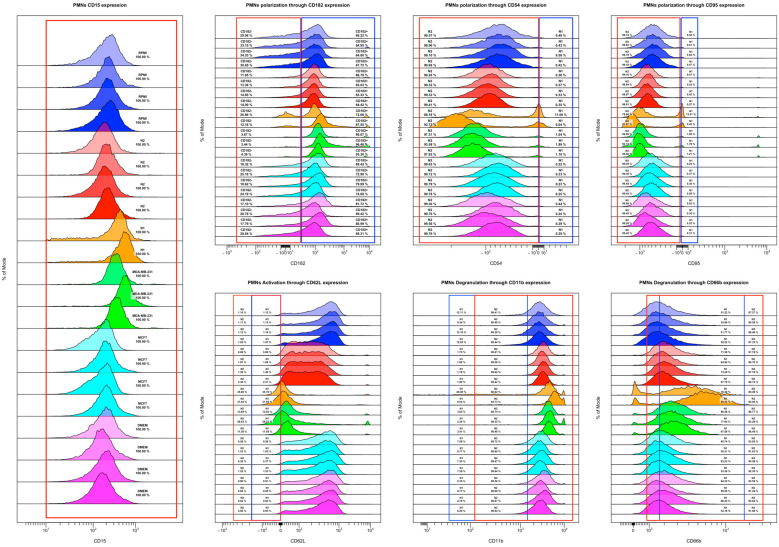
Frequency for each marker in the CD15+ population. Both N1 and MDA-MB-231 CM seem to exhibit similar inflammatory patterns on PMNs as opposed to other conditions. Blue = RPMI control, Red = N2, Orange = N1, Green = MDA-MB-231, Cyan = MCF7, Purple = DMEM control.

Using all markers in combination to assess PMN following CM treatment had shown that the MDA-MB-231 CM and N1-polarization cocktail had similar effects on cells. However, assessing the overall marker expression regardless of the subtype showed clear shifts between cell populations (Figure 10). Histogram visualization showed two peaks in CD182, CD54, CD95, and CD66b in the N1-polarized population. Interestingly, MDA-MB-231 CM peaks followed the N1-polarized peaks closely, with both N1 and MDA-MB-231 cells having a low expression of CD62L, which indicates that most of the neutrophils were activated. Furthermore, in N2, the CD62L peak is wide, indicating varying amounts of CD62L expression in its cell population. MCF-7 cells exhibited higher levels of CD62L expression compared to both N1 and N2 polarized cells, which further indicates that MCF-7 did not have such a high inflammatory effect on neutrophils.

**Figure 11:**
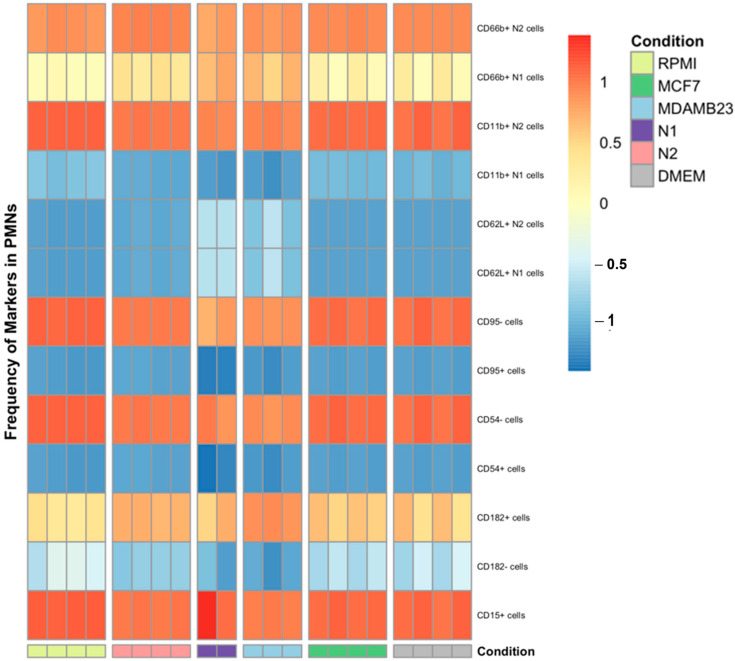
Heatmap of the frequency of the markers in PMNs.

Comparing the frequency of these PMNs showed clear changes in specific markers and populations (Figure 11). Sample frequencies were clustered according to the similarity of their expressions, and both N1 and MDA-MB-231 clustered together with differences in their activation, so that both conditions exhibited lower CD62L expression compared to other groups.

### 3.6. The Composition of the Polarization Cocktail and CM Plays a Role in Myeloperoxidase and Neutrophil Elasstase Production by dHL60 Cells

To understand the effect of the polarization cocktail and CM on neutrophils, we measured the levels of myeloperoxidase (MPO) and neutrophil elastase (NE) in the supernatant of treated dHL60 cells with BC CM or N1 and N2 polarizing cocktails.

**Figure 12:**
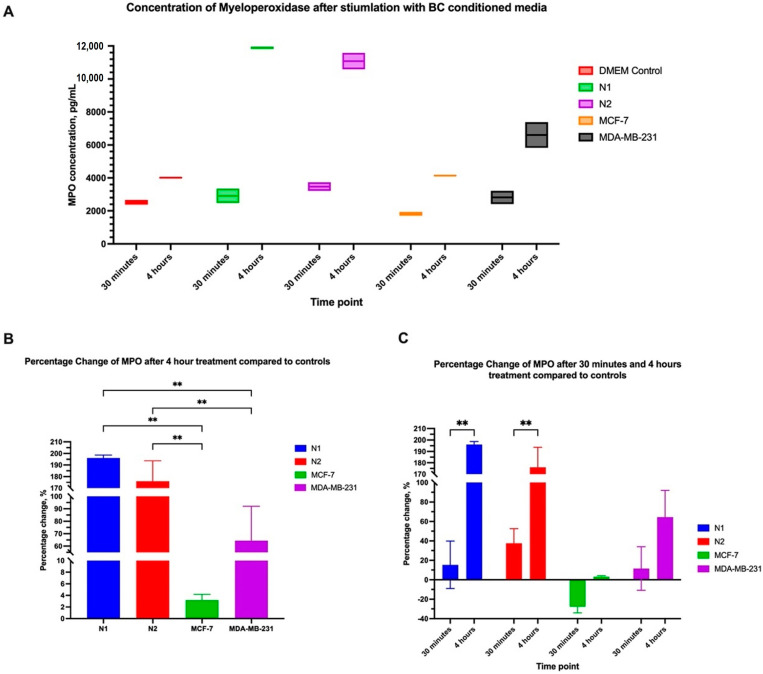
MDA-MB-231 CM increases myeloperoxidase concentration in the media. (**A**) concentration of MPO in the supernatant after 30 min and 4 h of stimulation with BC CM. (**B**) Percentage increase in MPO levels after 4 h of treatment compared to controls. (**C**) Comparing the percentage increase in MPO levels between 30 min and 4 h shows that N1 and N2 polarization cocktails had increased concentration by more than 100% between 30 min and 4 h. The statistical significance was calculated using one-way ANOVA and *t*-test and is denoted by ** = *p* < 0.01.

Myeloperoxidase is an enzyme that plays a role in neutrophil function by regulating inflammation and generating reactive oxygen species (ROS). Elevated levels of MPO are used as markers for inflammatory diseases, and in some cancers, are believed to aid in tumour progression [37]. dHL60 cells treated with BC CM had different levels of MPO expression between 30 min and 4 h. In the first 30 min of exposure to stimulants, there was no statistical difference in MPO levels, though MCF-7 CM-treated dHL60 had the least MPO in the supernatant (Figure 12).

After 4 h of stimulation, a statistical difference between the conditions was observed, with N1 and N2-polarized cells having a more than two-fold increase in MPO levels compared to the control. Furthermore, MDA-MD-231 CM had significantly elevated levels of MPO (x = 6603 pg/mL) compared to the control but had lower MPO levels compared to N1- (x = 11,890 pg/mL, *p* = 0.0002) and N2-polarized cells (x = 11,084 pg/mL, *p* = 0.0005). MCF-7 CM did not elevate MPO levels and was around the same concentration as the control sample (Figure 12B). Comparing samples between 30 min and 4 h showed that N1 and N2 had the most significant difference and increase in MPO expression in the media, with more than a 100% increase in MPO levels. MDA-MB-231 CM MPO expression also increased between 30 min and 4 h, but the increase was not statistically significant (Figure 12C). These findings indicate that prolonged exposure to inflammatory stimulants can increase MPO levels in the environment.

NE is another enzyme released by neutrophils to aid in the immune response against infection and is involved in tissue remodelling and cell migration. In the context of disease and cancer, this enzyme can help in tumour progression by activating pro-tumour neutrophils or inhibiting the anti-tumour immune response. Elevated levels of NE in cancer have been linked with later stages and more aggressive cancer, which in turn results in a poor prognosis [38].

**Figure 13:**
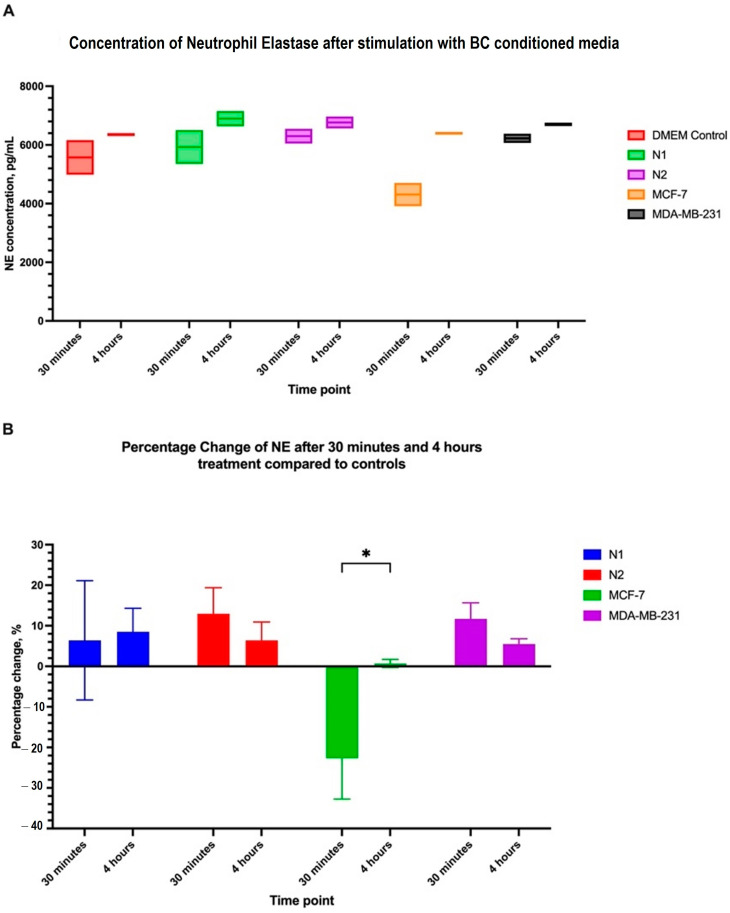
Neutrophil elastase in the microenvironment is not affected by BC CM. (**A**) The concentration of NE in the supernatant after 30 min and 4 h of stimulation with BC CM or polarizing cocktails. (**B**) Comparing the percentage increase in NE levels between 30 min and 4 h shows that MCF-7 had slightly elevated NE levels in the medium. The statistical significance was calculated using *t*-test and is denoted by * = *p* < 0.05.

There was no difference in NE levels in the supernatant of the dHL60 cells stimulated with BC CM compared to controls after 30 min and 4 h of stimulation. In the first 30 min, MCF-7 CM caused a non-significant reduction in NE levels (d = 1264 pg/mL ± 609.8; *p* = 0.3531) (Figure 13B). Additionally, only MCF-7 CM displayed a significant change in NE concentration between 30 min and 4 h, with a percentage increase of 23.4% (*p* = 0.0480), suggesting that MCF-7 CM may require some time to act on neutrophils in terms of NE generation (Figure 13A).

It is interesting to understand the cells that displayed such behaviours following stimulation, so we assessed the components of the polarization cocktail individually or with MDA-MB-231 CM. The N1 cocktail comprises LPS, interferon beta, and interferon-gamma, all of which regulate the immune response, specifically towards pathogens. The N2 cocktail is composed of L-lactate, Adenosine, C-GSF, IL10, PGE2, and TGF-Beta, all of which play a role in regulating and potentially inhibiting the proinflammatory response [19,25,39,40,41,42,43].

**Figure 14:**
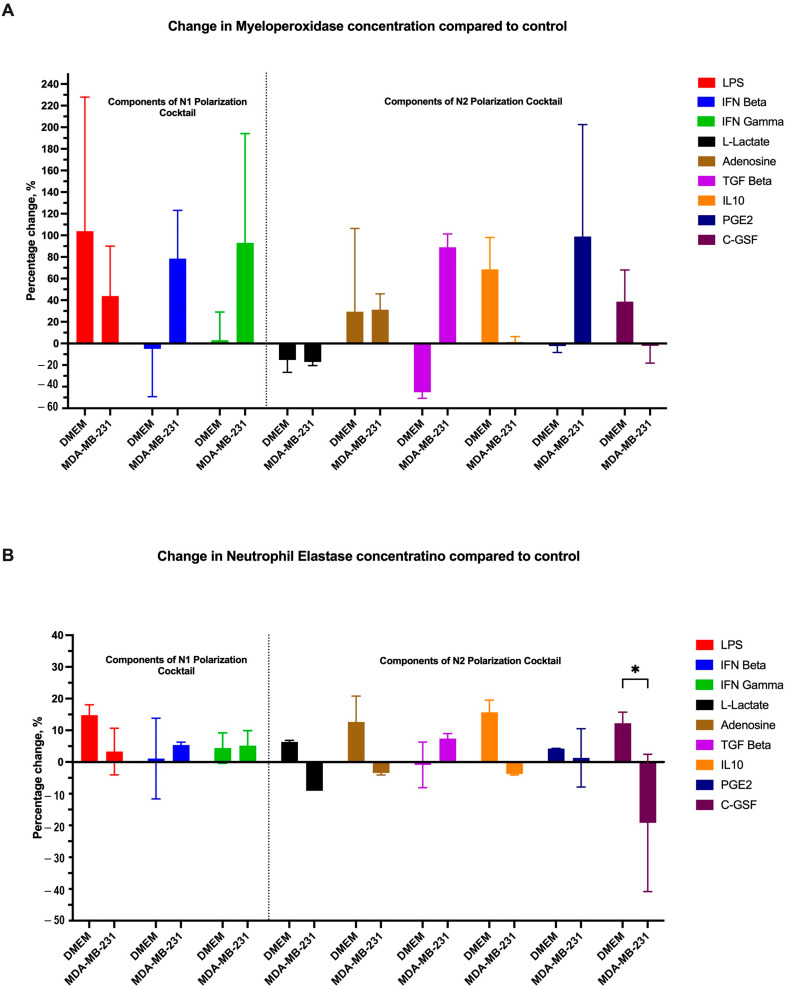
The differential effects of CM on different polarizing agents affecting the MPO and NE levels in the supernatant. (**A**) MPO was increased in the presence of CM, while (**B**) NE showed a different pattern with the most significant change with C-GSF. The statistical significance was calculated using *t*-test and is denoted by * = *p* < 0.05.

These components acting individually on MPO have varying effects based on the use of either growth media or MDA-MB-231 CM. In normal DMEM, among N1-cocktail components, only LPS could increase MPO concentration in the supernatant. The presence of MDA-MB-231 CM supplemented with each component increased MPO concentration in all conditions regardless. This suggests that MDA-MB-231 CM could increase the concentration of MPO more than using the polarization cocktail components individually. While in the N2 cocktail, using normal DMEM, L-lactate, and TGF-beta decreased MPO production, while PGE2 had no effect. Adenosine and C-GSF increased MPO concentration. The addition of MDA-MB-231 CM altered this effect in some conditions; for example, with TGF-Beta, there was a reversed effect between DMEM and MDA-MB-231 CM, with increased production of MPO with MDA-MB-231 CM. On the other hand, MDA-MB-231 CM inhibited the effect of IL-10, an anti-inflammatory and immunosuppressive cytokine, reducing MPO production (Figure 14A).

Regarding the effect on neutrophil elastase, all N1 polarization agents, regardless of BC CM’s presence, increased NE concentration in the supernatant. Interestingly, NE production in response to LPS was less in MDA-MB-231 CM compared to DMEM media. However, N2 polarization agents had different effects, with most increasing the concentration of NE. Adding MDA-MB-231 CM to L-lactate, adenosine, IL10, and CGSF, resulted in lower concentrations of NE in the supernatant. Only C-GSF had any significant difference in NE concentration when a normal medium or MDA-MB-231 CM was used (Figure 14B).

### 3.7. MDA-MB-231 CM Increased CD54 Expression on dHL60 and Polarized Them towards an Inflammatory Phenotype Similar to PMNs

**Figure 15:**
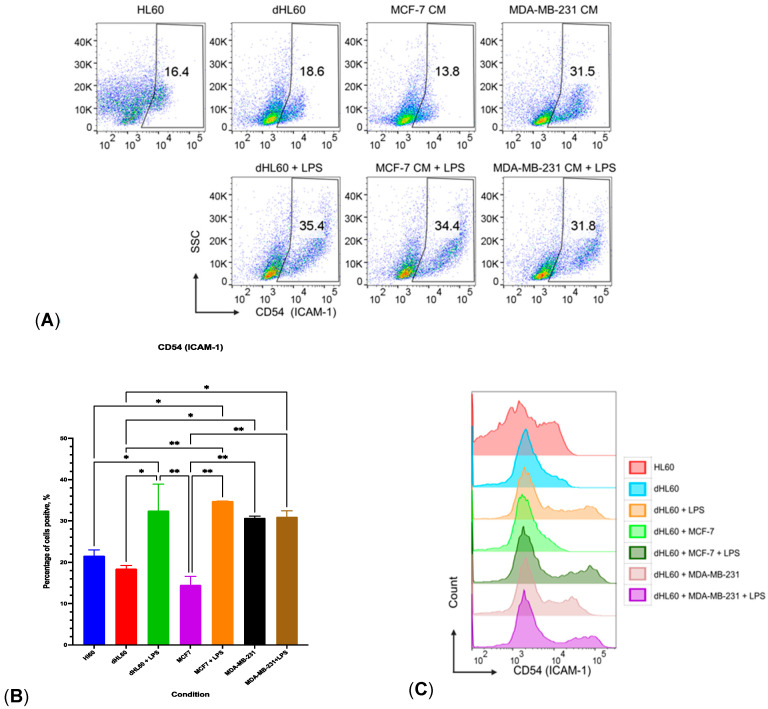
MDA-MB-231 CM increased CD54 (ICAM-1) expression on dHL60. (**A**) Flow cytometry dot plots show the percentage of cells having CD54 high expression. (**B**) Bar plot shows CD54 expression on neutrophil-like cell line HL60 followed by different treatments with BC CM with/without 100 ng/mL LPS stimulation. (**C**) Histogram plots show the expression of ICAM-1 with different treatments. The statistical significance was calculated using one-way ANOVA and is denoted by ** = *p* < 0.01; * = *p* < 0.05.

Recently, it was shown that a subset of neutrophils, which is CD54^High^, has proinflammatory properties but a decreased chemotactic response, in contrast to CD54^Low^ cells, which have decreased proinflammatory properties and higher chemotaxis [44]. Based on that, and the fact that LPS was the only polarizing agent to increase MPO and NE production regardless of the media used, we assessed the effect of BC CM and LPS on the CD54 expression of dHL60. Results revealed that 24-h treatment with CM from MDA-MB-231 increased the levels of CD54 significantly compared to MCF-7 (Figure 15). It was also evident that LPS alone increased CD54 expression by 35.4% on dHL60 cells compared to untreated controls (18.6%). MCF-7 CM did not affect CD54 expression (13.8%); however, the addition of LPS increased the expression of CD54 to 34.4%.

Interestingly, MDA-MB-231 CM alone increased CD54 expression significantly (31.5%), while adding LPS did not cause an additional increase in CD54 expression (31.8%). This high CD54 expression can be indicative of polarization towards a proinflammatory phenotype. Since CD54 expression was similar to that of LPS stimulation, collectively, our results demonstrate that molecules in the MDA-MB-231 CM triggered dHL60 cells towards a proinflammatory N1 phenotype like LPS. Furthermore, this shows that dHL60 can exhibit properties comparable to neutrophils isolated from whole blood.

### 3.8. dHL60 Showed a Varying Trans-Well Migration in Response to MCF-7 CM and MDA-MB-231 CM

**Figure 16:**
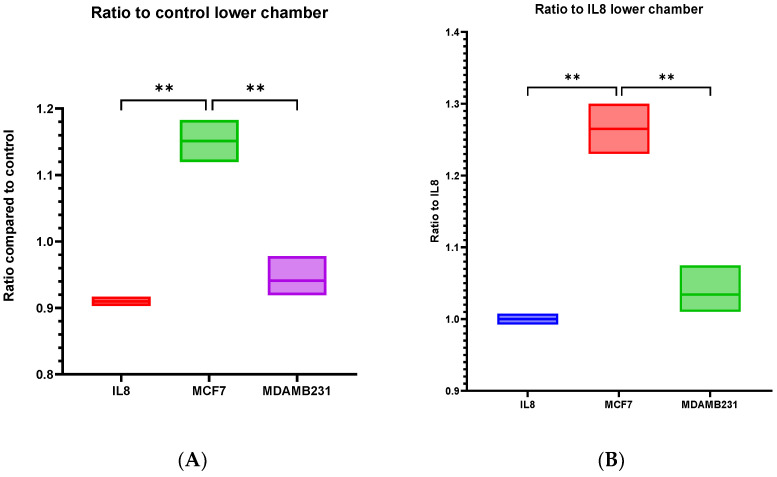
The ratio of cells that have migrated to the lower chamber in dHL60 trans-well migration assay in response to IL8 or BC CM. (**A**) Comparing the cell ratio to that of DMEM control, and (**B**) comparing the cell ratio to the positive control, IL8. The statistical significance was calculated using one way ANOVA and *t* test and is denoted by the following: ** = *p* < 0.01.

The chemo-attractive capabilities of BC CM were assessed using a trans-well migration assay. dHL60 cells were incubated in the upper chamber while the lower chamber was supplemented with normal culture medium (as negative control), IL-8 chemoattractant (as positive control), or CM of both MCF-7 and MDA-MB-231, then incubated at 37 °C for one hour and then were quantified using NucBlue and Giemsa stain. The chemo-attractive capacity of MDA-MB-231 CM on dHL60 was comparable to the IL8 (a well-established chemoattractant of neutrophils) (Figure 16A,B).

**Figure 17:**
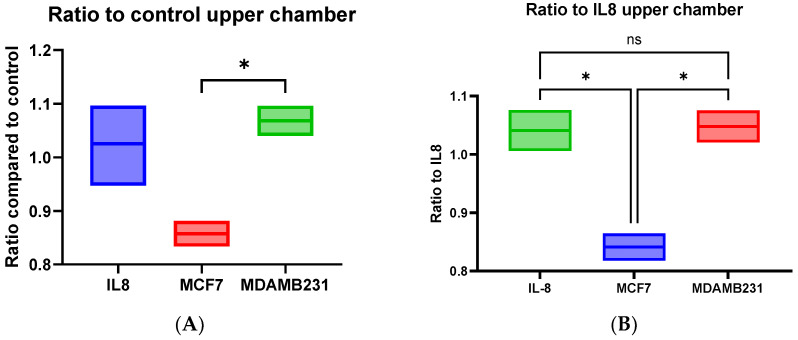
The ratio of cells that have not migrated and remain in the upper chamber in dHL60 trans-well migration assay in response to IL8 or BC CM. (**A**) Comparing the cell ratio to that of DMEM control, and (**B**) comparing the cell ratio to the positive control, IL8. The statistical significance was calculated using one-way ANOVA and *t* test and is detonated by the following: * = *p* < 0.05, ns = not significant.

Cells migrate towards the lower chamber in response to stimuli, and MCF-7 CM caused the greatest number of cells migrated towards the lower chamber. MDA-MB-231 CM elicited a response similar to IL8, as there are no statistical differences between the two conditions. The estimation of cells remaining in the upper chamber was also similar, with MDA-MB-231 CM causing cells to be less in the upper chamber (Figure 17A,B). Possible factors that could contribute to this lack of response were incomplete polarization and altered expression of MPO and NE.

**Figure 18:**
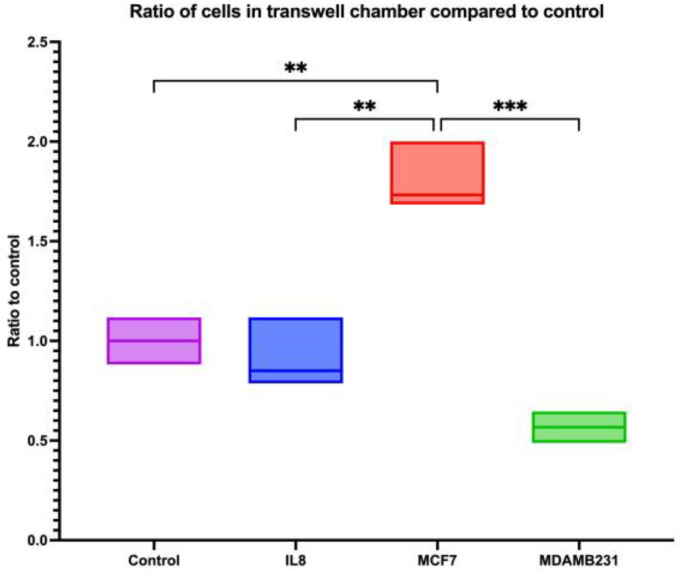
The ratio of cells that remained in the trans-well chamber in dHL60 trans-well migration assay in response to IL8 or BC CM. The ratio of dHL60 cells remaining in the trans well membrane compared to controls. The statistical significance was calculated using one way ANOVA and *t* test and is detonated by the following: *** = *p* < 0.001; ** = *p* < 0.01.

Giemsa-stained cells reflect the migration through the trans-well chamber, revealing that MCF-7 CM stimulated cells had the highest ratio of cells compared to controls and IL8. Ratio comparison to controls showed a statistically significant difference between MCF-7 CM and controls (*p* = 0.0066), IL8 (*p* = 0.0028), and MDA-MB-231 CM (*p* = 0.0010). When comparing them to IL8, the mean difference between MCF-7 and IL8 was 0.9657 (*p* = 0.0023), and MCF-7 CM with MDA-MB-231 CM was 1.349 (*p* = 0.0012) (Figure 18).

NucBlue is a nuclear stain for living cells, and low expression of this stain can indicate cells undergoing cellular and/or nuclear changes, such as those seen in NETosis. As seen previously, MDA-MB-231 CM can induce NETosis as early as 30 min upon stimulation, and as such, dHL60 cells might have undergone a form of cell death. This can explain why MDA-MB-231 CM stimulated cells had similar cells migrated towards the lower chamber as the controls, but fewer cells remained in the upper chamber and within the trans-well layer. This indicates that BC CM contains cytokines that can affect the recruitment and migration of neutrophils to the local environment.

### 3.9. CD11b Was Induced More in dHL60 Co-Cultured with MDA-MB-231

**Figure 19:**
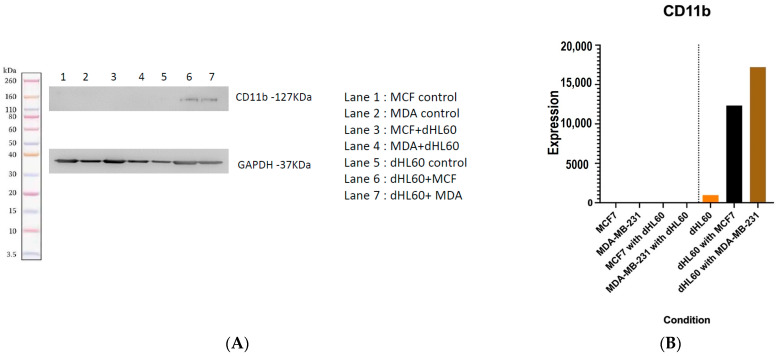
Western blot of CD11b with GAPDH used as a housekeeping protein. (**A**) CD11b bands appear around 127 kDa, being the most significantly expressed protein. The uncropped blots are shown in the supplementary materials. (**B**) ImageJ software (ImageJ v1.53) quantification of the protein shows that BC cell lines increased the expression of CD11b by dHL60 cells.

CD11b is a receptor that exhibits increased expression in degranulating neutrophils and is associated with migration of neutrophils [45]. Higher CD11b expression on cells indicates higher levels of granules in neutrophils. In flow cytometry results, only surface-expressed CD11b was measured, as antibodies are cell surface antibodies and bind towards specific regions of the receptor; therefore, modified or intracellular receptors are not measured by flow cytometry. Here, cells were co-cultured, and cell lysate was used for western blotting analysis, which showed that CD11b was not expressed on BC cell lines, as it is primarily expressed on leukocytes (Figure 19). dHL60 naturally expresses some degree of CD11b; however, following co-culture with BC cell lines, there was an increase in CD11b expression. This was shown by faint and thick bands appearing at around the 127 kDa marker. The slightly increased expression of CD11b was observed in dHL60 cells co-cultured with MDA-MB-231 cells compared to those co-cultured with MCF-7 cells (Figure 19). These findings are in line with the previously observed enhanced migratory capacity of dHL60 cells treated with MCF-7 CM. However, MDA-MB-231 CM did not elicit such a high migratory response, perhaps signifying that its chemotaxis capabilities have been affected by other factors secreted in the CM. Interestingly, the high levels of CD11b observed could be due to direct interactions between the two cell lines rather than the secreted chemokines. This finding adds to the complexity of the crosstalk between neutrophils and BC, where different factors alter how cells behave.

## 4. Discussion

This study provided several aspects of the crosstalk between BC cells and neutrophils to further understand the important role of neutrophils as an important component of the tumour microenvironment. We have demonstrated that BC cells or their CM can reduce the viability and the proliferation of neutrophil-like cells (HL60). This was supported by the increased cellular stress and NETosis in the dHL60 cells upon exposure to TNBC CM. We further illustrated, using flow cytometry analyses, the inflammatory markers profile of PMNs by comparing BC CM to established polarizing cocktails. It was found that TNBC CM triggered a more inflammatory pattern (comparable to N1-subtype) with evidence of stronger adhesion (CD62L) and the degranulation (CD11b and CD66b) phenotype. The proinflammatory polarization of dHL60 under the effect of TNBC CM was additionally confirmed by the elevated CD54 expression, increased MPO production, and the overexpression of CD11b. The latter marker matched the observed increased migratory capacity of dHL60 cells in the trans-well migratory assays.

Polymorphonuclear neutrophils (PMNs) originate from the bone marrow during haematopoiesis in response to various cytokines released in response to stimuli such as inflammation [11,46,47]. The cell line HL60 is used as a model for human neutrophils. DMSO and/or ATRA stimulation differentiate these cells into a more neutrophil-like state and exhibit neutrophil-like properties. This change in cellular morphology has been visualized with the Giemsa stain, as it has been suggested that DMSO acts by inducing genomic changes by upregulating certain genes that are involved with differentiation and granulation. Furthermore, flow cytometry data show some differences in FSC-A and SSC-A between HL60 and dHL60. These distinct changes in cell size offer a more homogenous population of cells in dHL60 than HL60 [48].

### 4.1. Cell Viability Changes as Crosstalk Variable between BC and Neutrophils

We have demonstrated that BC cells or their CM can reduce neutrophil-like cells’ viability and proliferation (dHL60). Our data show that dHL60 is more effective at inhibiting breast cancer proliferation compared to the cells in their non-differentiated state, as well as that increased concentrations of CM had a more significant effect. On the other hand, BC-CM is also able to inhibit the proliferation and reduce the viability of dHL60 cells, with this inhibition being time- and dose-dependent. This indicates that BC acts on neutrophils in different life cycle stages and response rates vary according to the concentration of CM.

The co-culturing of MDA-MB-231 (high metastatic) and MCF-7 (weakly metastatic) at different stages of their adhesion with dHL60 showed different responses. BC cells that were cocultured before adhesion to the plate had no difference in their viability. However, dHL60 viability decreased when cocultured with MDA-MB-231, which is in line with other studies that had shown that MDA-MB-231 cells polarize neutrophils towards a pro-tumour subtype [48]. The lack of decrease in viability could be attributed to MDA-MB-231 acting as circulating tumour cells (CTCs), which require neutrophils to expand their metastatic potential [49]. On the other hand, MCF-7 cell lines exhibit decreased neutrophil viability in post-adherence due to the low release of certain soluble factors like chemokines contributing to neutrophil infiltration [49].

### 4.2. NETosis and Phenotype Modification Is Another Aspect of BC and Neutrophils Crosstalk

Other changes in neutrophil activity could be attributed to the different mechanisms PMNs use to destroy pathogens. One mode of action is the depositing of NETs, and these traps are utilized by the process of NETosis, resulting in cell and pathogen death [8,9,10,11]. However, NETs have been implicated in the potential of metastasis mediation [12], where increased levels of NET formation are linked to tumour progression and metastasis of several malignancies [13,14]. In our study, this was supported by the increased cellular stress and NETosis in the dHL60 cells upon exposure to TNBC CM, as seen in our results where MDA-MB-231 CM caused a similar rate of NETosis as that of LPS-induced dHL60 NETosis.

We further illustrated the crosstalk through flow cytometric analysis of the inflammatory marker profile of PMNs and comparing the effect of BC CM to that of established polarizing cocktails. TNBC CM triggered a more inflammatory pattern (comparable to N1-subtype) with evidence of stronger adhesion (CD62L) and the degranulation (CD11b and CD66b) phenotype. The elevated CD54 expression additionally confirmed the proinflammatory polarization of dHL60 under the effect of TNBC CM, increased MPO production, and the overexpression of CD11b.

Additionally, during NETosis, neutrophils lose some of their characteristic features and become more irregular in shape [50], and therefore, when assessing the results using flow cytometry, it is difficult to identify and gate cells accurately. They also form cell aggregates and clumps, which prevent the single cell readings, which is a negative effect when using a flow cytometer. Furthermore, the release of NETs and other cytoplasmic products into the media might interfere with the fluorescent signals and the binding capabilities of antibodies used to detect specific neutrophil markers, therefore affecting the sensitivity of the assay.

### 4.3. BC and Neutrophil Crosstalk Enhances the Releases of Substances That Aid Tumour Progression

Furthermore, HL60/dHL60 neutrophil cellular model has shown capabilities to somehow polarize into one of the two tumour-associated neutrophil (TANs) populations. The use of BC CM and polarizing agents has shown changes to the expression of some cytokines and chemokines. For example, the addition of MDA-MB-231 (TNBC) CM increased CD54 (ICAM-1) expression in dHL60, which is associated with the adhesion and migration of proinflammatory subtypes of PMNs [51,52]. Similar changes of markers caused by MDA-MB-231 CM and N1 polarization agents further support the notion that MDA-MB-231 can elicit inflammatory responses [19].

Studies cited that coculturing neutrophils with a highly metastatic (MDA-MB-231) and a weakly metastatic (T47D) BC cell line, or the exposure to their CM, results in the production of oncostatin M, using GM-CSF secreted by breast cancer [53] which in turn increases expression of vascular endothelial growth factor (VEGF) and increases the invasive properties of BC [53]. Furthermore, GM-CSF produced by MDA-MB-231 is responsible for neutrophil changes that drive tumour cells’ transmigration through the endothelial barrier and facilitate potential metastasis [54]. The extracellular vesicles produced by this cell line can polarize neutrophils towards the pro-tumour (N2) subtype, which increases the migratory potential, the release of NETs, extracellular DNA, IL-8, ARG-1 expression, MMP9 activity, and the overall increase in viability of TNBC cells [48].

ELISA has shown that MDA-MB-231 CM increased MPO and NE levels after 4 h, and that increase in MPO can be one of the factors to aid in their progression and metastasis. MPO is believed to be close to the mechanism of NETosis and related nuclear and cellular changes leading to it [55]. On the other hand, MCF-7 inhibited the production of MPO and NE, and it did not exhibit any changes or increases in CD54 levels, all of which can be attributed to its low metastatic properties.

Western blotting analysis revealed increased expression of CD11b in dHL60 cocultured with BC cells. Both MCF-7 and MDA-MB-231 increased the expression of this surface marker in neutrophils upon coculturing. CD11b is linked to cellular adhesion, and its expression is increased in the exocytosis of tertiary granules [56]. Studies have shown that increased CD11b expression in neutrophils is linked with angiogenesis by releasing and increasing the expression of VEGF and MMP9 [57]. Other studies had reported that upon treatment of neutrophils with the CM of a poorly metastatic BC cell line (MDA-MB-468), the expression of CD11b/CD18 was upregulated due to the CXCL1 and IL-8 that is secreted by this cell line [58].

### 4.4. Limitations and Future Directions

This study provided several aspects of the crosstalk between BC cells and neutrophils to further understand the important role of neutrophils as an important component of the tumour microenvironment. While different in-vitro techniques and approaches were utilized to examine variable aspects of the crosstalk between BC and neutrophils in this report, there are inherent limitations of this design. First, in vivo models to validate the results using TNBC xenograft animals and to evaluate neutrophil infiltration and differentiation is warranted. Second, the reported experiments can be replicated on other BC cell lines—more specifically, different types of TNBC cell lines to provide further understanding about the progression and aggressiveness of BC types. Third, throughout the report, we have highlighted unavoidable bias in the results due to the technical specification of the experiments such as the cellular differentiation and flow cytometry outcomes in clumped cells. Furthermore, while the use of HL60 to model neutrophils is an ideal alternative to using neutrophils, there are many factors, such as the complex immune microenvironment and the life cycle of immune cells, which can all affect the ways these cells behave towards certain stimuli [59,60].

The exploration of the TANs role In TNBC progression and the associated microenvironment interactions can help in improving immunotherapy strategies such as NETosis inhibition and the neutrophil N1/N2 phenotype switch. Further analysis of the chemotaxis properties of the BC CM, which were found comparable to well-established polarizing agents, can identify novel therapeutic targets, counteracting the TANs protumour effect or guide proper combination therapies. In addition, we have illustrated the overexpression of specific inflammatory markers by neutrophils upon BC coculture of CM exposure (such as MPO, NE and CD11b). These molecules are implicated in tumour progression, and hence they add up to the list of potential therapeutic targets to control the tumour microenvironment. Several studies have been conducted on the use of these molecules as therapeutic targets, and some are undergoing clinical trials [15,61].

## 5. Conclusions

Breast cancer is a complex and heterogeneous disease that affects more than 2 million females worldwide. While the disease affects older women, in recent years, there has been an increase in incidence rates among younger women. Various factors affect the disease, including its vast immune microenvironment.

In vitro validation showed that TNBC-CM can stimulate and activate CD54 in neutrophils and induce NETosis in the dHL60 cell line. Additionally, coculturing of MDA-MB-231 and dHL60 at the seeding stage reduced the viability of dHL60, which is an indicator of cell death by various means, such as NETosis. Furthermore, CD11b expression increased in dHL60 cocultured with BC cells; however, not all effects were visualized, which paints the differences between direct and indirect cellular signalling.

To conclude, neutrophils play a role in tumour prognosis and survival, as in vitro tests reveal increased CD11b, CD54, and CD66b expression when coculturing neutrophils with BC cell lines or their conditioned media. Furthermore, BC cells use neutrophils to their benefit through NETosis, cytokines, and chemokines. This cross-talk between BC and neutrophils needs to be further investigated, as it could decipher the mechanisms by which TNBC uses the immune system to its benefit.

## Figures and Tables

**Table 1:** N1 and N2 polarization cocktails and their stimulatory roles in the microenvironment.

**N1 Cocktail**	**Stimulatory Role**
LPS	Proinflammatory: a bacterial product that induces proinflammatory cytokines and interacts with TLR4 [19,20].
IFN Beta	Proinflammatory: an antiviral cytokine that enhances polarization towards N1 phenotype [19,21].
INF Gamma	Proinflammatory: Regulatory cytokine that is linked with neutrophil activation [19,22].
**N2 Cocktail**	**Stimulatory Role**
L-Lactate	Anti-inflammatory: Mimics tumour microenvironment as it is a by-product of cellular metabolism [19,23].
Adenosine	Anti-inflammatory: Mimics tumour microenvironment and is an immunosuppressive molecule [19,24].
TGF Beta	Anti-inflammatory; immunosuppressive regulatory cytokine [19,25].
IL-10	Anti-inflammatory; Cytokine with immune inhibitory functions [19,26].
PGE2	Anti-inflammatory lipid mediator that inhibits the production of proinflammatory cytokines [19,27].
C-GSF	Produces granulocytes in neutrophils [19,28].

## Data Availability

All data are presented in the paper.

## Supplementary Materials

The following supporting information can be downloaded at: https://www.mdpi.com/article/10.3390/cancers16040747/s1, Full pictures of the Western blots.
