# Supplementary material for: Characterizing the Inflammatory Profile of Neutrophil-Rich Triple-Negative Breast Cancer
Source: Cancers (Basel). 2024 Feb 10;16(4):747. doi: 10.3390/cancers16040747 (PMC10886617; doi:10.3390/cancers16040747)
Supplement: Supplementary file 1 [file cancers-16-00747-s001.zip › cancers-2763276-supplementary.pdf]

Co-Culture_Western Blot_CD11b

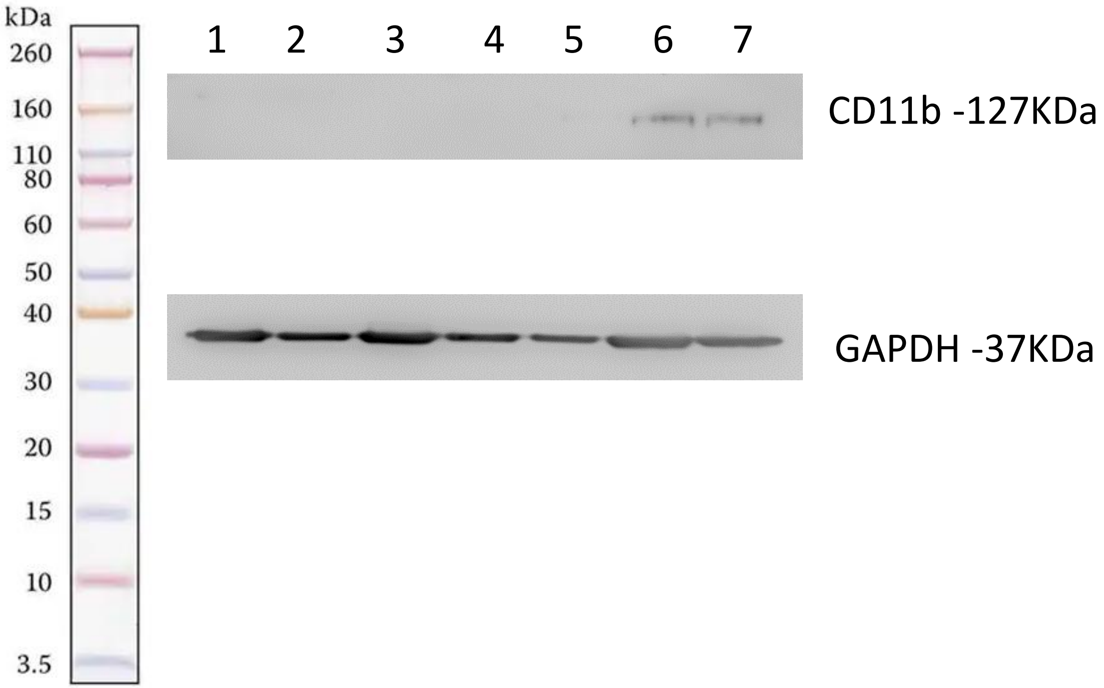

Lane 1 : MCF control
Lane 2 : MDA control
Lane 3 : MCF+dHL60
Lane 4 : MDA+dHL60
Lane 5 : dHL60 control
Lane 6 : dHL60+MCF
Lane 7 : dHL60+ MDA
Note: There was a faint band in dHL60 control but not visible in image

Co-Culture_Western Blot_CD11b

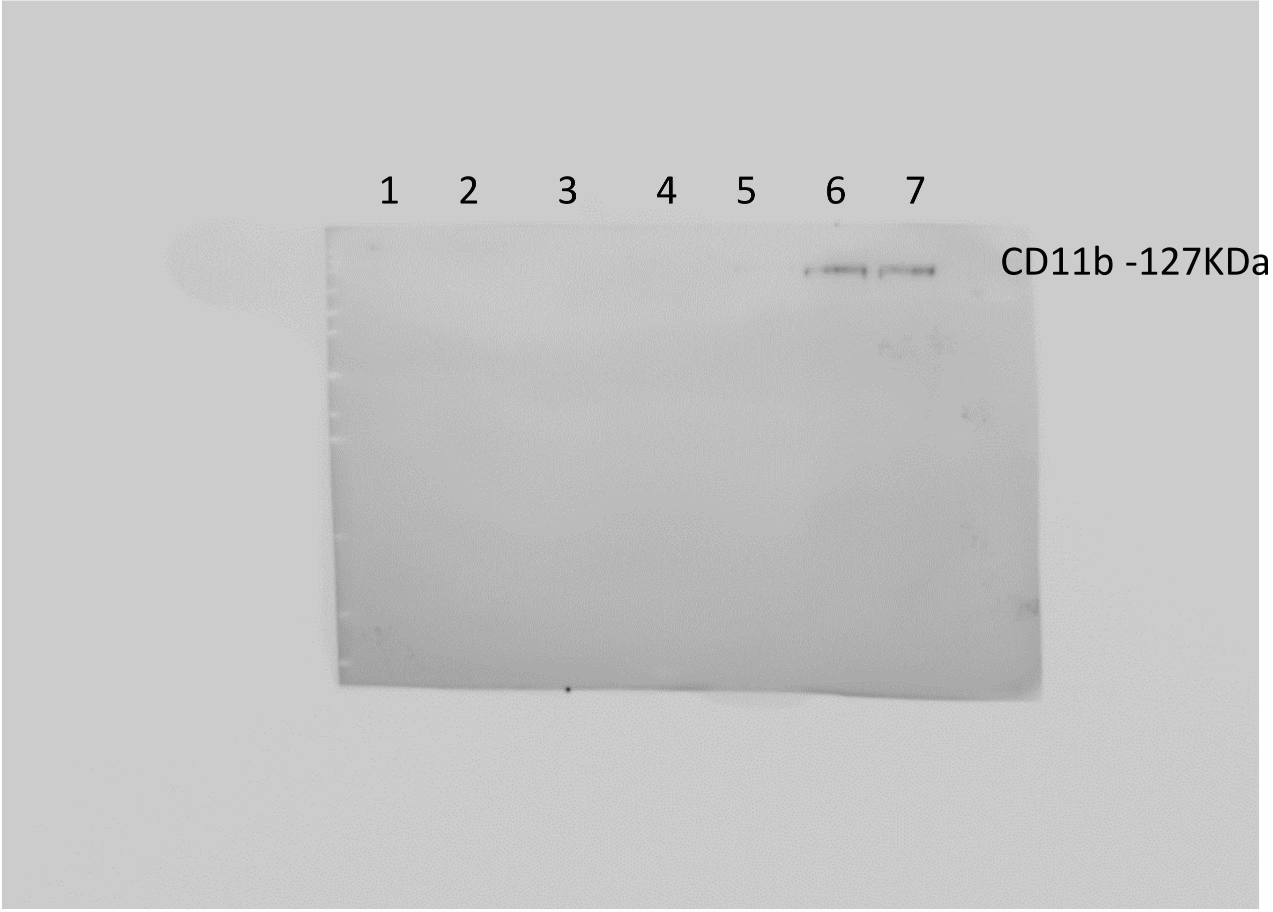

Lane 1 : MCF control
Lane 2 : MDA control
Lane 3 : MCF+dHL60
Lane 4 : MDA+dHL60
Lane 5 : dHL60 control
Lane 6 : dHL60+MCF
Lane 7 : dHL60+ MDA
Note: There was a faint band in dHL60 control but not visible in image

Co-Culture_Western Blot_CD11b

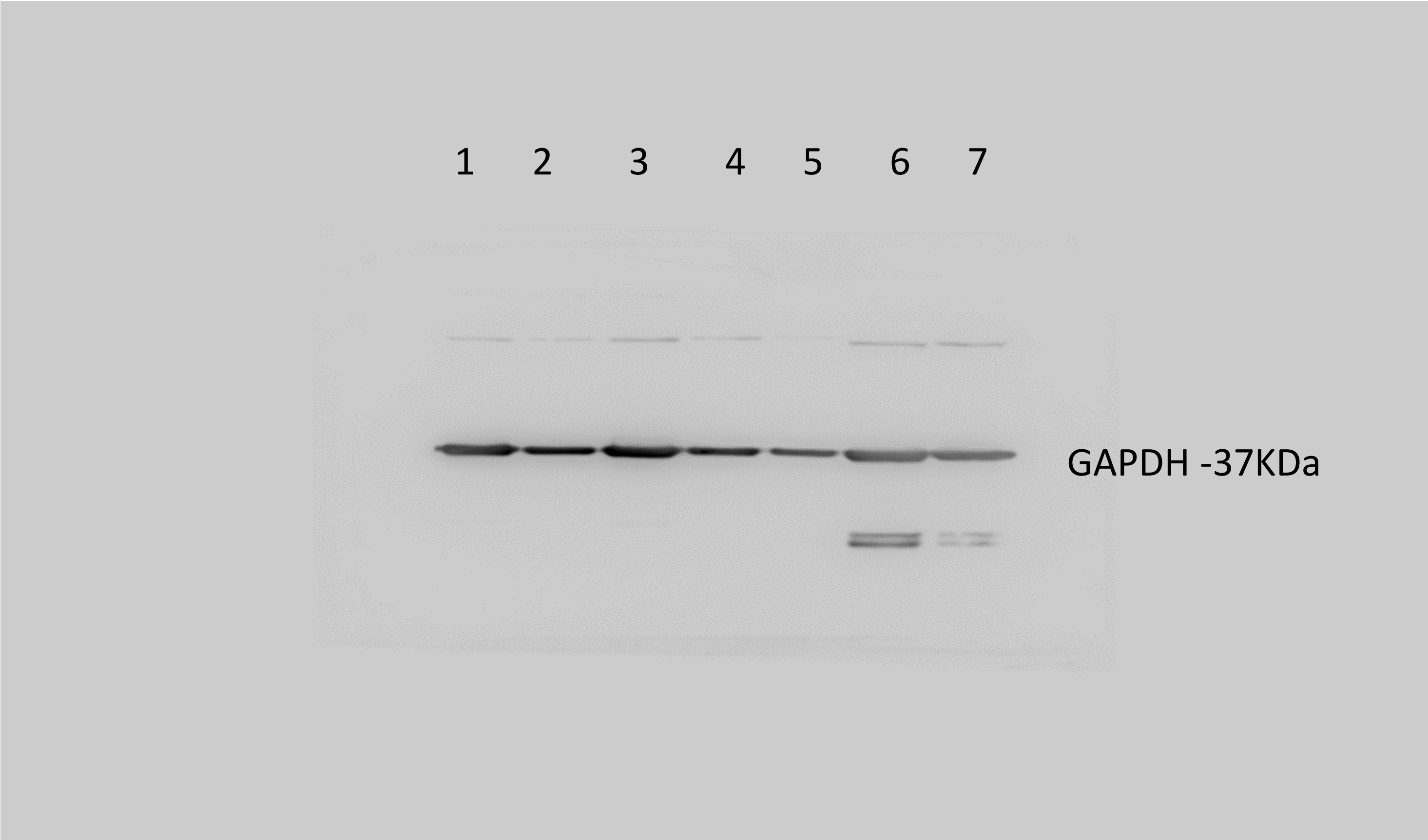

Lane 1 : MCF control
Lane 2 : MDA control
Lane 3 : MCF+dHL60
Lane 4 : MDA+dHL60
Lane 5 : dHL60 control
Lane 6 : dHL60+MCF
Lane 7 : dHL60+ MDA
Note: There was a faint band in dHL60 control but not visible in image
